# Ergospirometry with concurrent fibre optic laryngoscopy: a randomised crossover study

**DOI:** 10.1080/20018525.2017.1399033

**Published:** 2017-11-20

**Authors:** Kiran Kafila Mirza, Emil Schwarz Walsted, Vibeke Backer

**Affiliations:** ^a^ Respiratory Research Unit, Department of Respiratory Medicine, University Hospital Bispebjerg, Copenhagen, Denmark

**Keywords:** Exercise-induced laryngeal obstruction, EILO, exercise continuous laryngoscopy during exercise, peak oxygen uptake, maximum oxygen uptake

## Abstract

**Background**: Patients suffering from exercise-induced laryngeal obstruction (EILO) are subjected to several exhausting tests. We aimed to assess the feasibility of using a single test to obtain diagnostic measurements for maximum oxygen uptake (VO_2_max) and exercise-induced laryngeal obstruction (EILO).

**Methods**: Patients referred to the outpatient respiratory clinic at the University Hospital of Bispebjerg, Copenhagen with exercise-induced dyspnoea were evaluated for inclusion over 13 months. Eligible patients were aged 18–43 years, had a known EILO diagnosis (moderate or severe) and were inactive (self-reported activity) with less than 3 hours activity per week. In randomised order, all participants (*n *= 11) underwent three tests: a VO_2_max test with and without concurrent laryngoscopy. VO_2_max and EILO values from the two testing methods were compared.

**Findings**: There was no difference in VO_2_max measured by ergospirometry with and without simultaneous continuous laryngoscopy during exercise (CLE) testing (mean difference −22 ml O_2_･min^−1^; 95% CI −125 to 81 ml O_2_･min^−1^; *P *= 0.647). EILO scores obtained during the CLE testing on the treadmill versus CLE testing on the ergometer bike revealed identical supraglottic scores in nine of the 11 participants (82%) with substantial agreement between the two types of test (*x* = 0.71). Glottic scores were identical in six of the 11 (55%), showing moderate agreement between test types (*x* = 0.38).

**Conclusions**: Based on our findings in inactive individuals, ergospirometry with laryngoscopy is feasible and well tolerated, yielding measurements for maximal oxygen uptake comparable to those of standard bike ergospirometry. Likewise, measurements of supraglottic EILO are comparable to those of the standard treadmill CLE test.

## Introduction

Assessment of exercise responses provides critical information when evaluating patients with, or suspected of having, cardiovascular or pulmonary diseases.[1] For example, maximal oxygen uptake (VO_2_max) obtained from ergospirometry has been described as ‘the single most influential concept in modern exercise physiology’.[2–3] Cardiopulmonary exercise testing is useful in evaluating exertional dyspnoea to distinguish between the different components of exercise limitation, i.e. to determine a patient’s fitness level [2] and detect pulmonary [4] or [5] cardiovascular exercise limitations.[6,7]

The extrathoracic airway is increasingly recognised as a potential source of exertional breathlessness.[8–10] Accordingly, continuous laryngoscopy during exercise (CLE) has emerged as a central test modality in investigating exercise-induced laryngeal obstruction (EILO), a transient narrowing of the airway. Visualisation of the larynx when a patient is symptomatic (i.e. during exercise) is crucial to detect EILO and to determine how the laryngeal structures are affected.[11,12] The clinician needs this detailed recording of laryngeal movement when evaluating a patient and particularly when assessing the possible benefit of interventions such as speech- and language therapy or surgery. Other techniques, such as inspiratory flow-volume curves,[13,14] bronchoprovocation testing [11,14,15] and flexible fibre optic laryngoscopy pre- and post-exercise,[9,16] do not provide reliable and accurate information about EILO. Thus CLE remains the de-facto gold standard for diagnosing EILO.

Combining the two tests (i.e. VO_2_max and CLE test) to yield diagnostic measurements for the ergospirometry (i.e. VO_2_max) and EILO in a single test would relieve patients of an additional test. However, the validity of oxygen uptake measurements recorded during ergospirometry with concurrent laryngoscopy has not been documented. Accordingly, we aimed to assess the diagnostic feasibility and patient acceptance of the two examinations performed simultaneously in patients with EILO. We hypothesised that oxygen uptake measurements obtained during ergospirometry with CLE would not differ from oxygen uptake measured with ergospirometry alone.

## Methods

**Figure 1.**
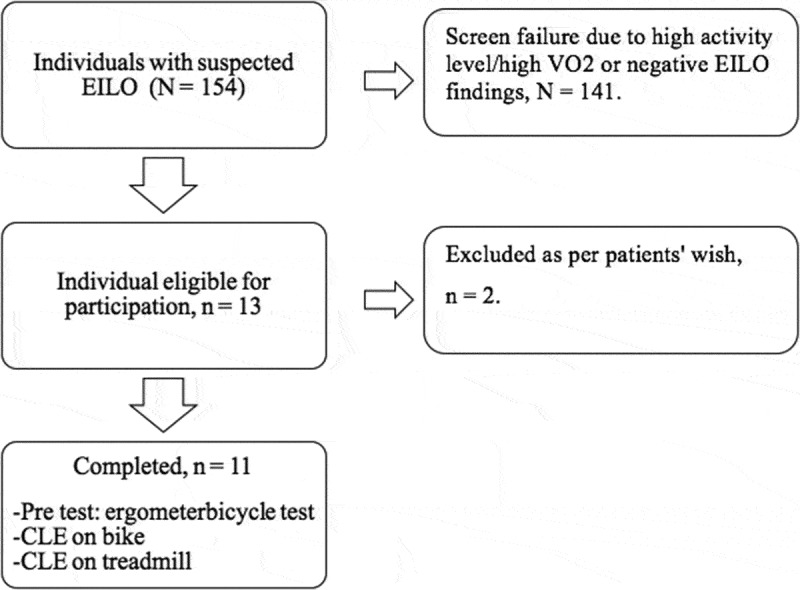
Flow diagram. All patients registered to undergo a CLE during the study period were considered for inclusion, *n *= 154 patients. Screen failures were due to negative EILO findings,[2] self-reported activity (daily workout > 3 hours or daily cycling commute longer than 15 km),[4] and high VO_2_max.[5] Thirteen patients were eligible for inclusion of whom two declined due to unrelated reasons. Eleven participants underwent all three tests, i.e. pre-test on ergometer bicycle test, continuous laryngoscopy during exercise (CLE) on ergometer bicycle and CLE on treadmill.

Patients were referred during April 2015–May 2016 to the respiratory outpatient clinic at the University Hospital of Bispebjerg, Copenhagen with exercise-induced shortness of breath (Figure 1). All participants underwent screening with double testing using ergospirometry and CLE tests on two separate days to determine the optimal ergospirometry test protocol for each patient. Eligible individuals underwent ergospirometry with and without simultaneous CLE testing in a randomised order using sealed envelopes. The three tests were performed within 7 days of each other.

The study was approved by the Danish Ethics Committee (H-4-2014-120) and all participants provided written informed consent.

### Inclusion and exclusion

We considered 13 patients for inclusion, of whom 11 were aged 18–43 years, had moderate to severe EILO and were inactive [17] (Figure 1).

Inclusion criteria were moderately fit patients without significant diseases other than EILO.

To ensure a homogenous group, exclusion criteria were self-reported level of physical activity of more than 3 hours per week [4] and a daily cycling commute longer than 15 km.[4] Any active infections or unstable conditions (including untreated cardiac diseases and untreated asthma) led to exclusion.

### Test preparations

Before all exercise tests, all participants received two puffs of salbutamol 0.2 mg. In addition, all participants received two nasal puffs of topical anaesthetic and xylometazoline spray before the CLE tests. Equipment was calibrated according to the manufacturer’s instructions.

### Procedures

#### CLE treadmill test

Initial diagnosis of EILO was made in accordance with Heimdal et al.,[18] using a flexible video rhinolaryngoscope and Visera video recording equipment (Olympus, Tokyo, Japan) during maximal treadmill exercise. The test was preceded by 8–10 min warm-up on the treadmill at an easy pace. CLE tests continued until complete exhaustion, at a self-determined constant speed on the treadmill with an increase of 3% in treadmill-elevation from time = 2 min and then every 1.5 min.[18]

All laryngoscopy recordings were performed by one of two examiners (K.M. and E.W.) and were individually graded at maximal exertion in accordance with Maat and colleagues [17] (i.e. none, mild, moderate, severe). Any score discrepancy not settled by discussion was settled by third-party review (V.B.). Eligible participants underwent the next test within 30 days of the CLE treadmill test.

#### Bike ergospirometry

Using the OxyCon Pro system, participants underwent a 5-min warm-up session followed by an incremental load test designed to reach peak exercise in 7–10 min.[7] Depending on the participant’s estimated physical condition, load started at 25 or 50 W, increasing 5 W every 30 seconds during the warm-up, followed by the test where the load was reset to 25 or 50 W, respectively. The load was increased by 2 W every sixth second until complete exhaustion was reached. Objective criteria were set for the tests [2,19,20] in order to audit the tests as described by Astorino,[2] Howley [19] and Midgley.[20]

#### CLE and bike ergospirometry

Using a flexible video rhinolaryngoscope through a modified mask (i.e. fillable passage in mask) all participants exercised on an ergospirometric bike (OxyCon Pro system); the test was preceded by a 5-min warm-up session followed by an incremental load test [21] with settings determined by the pre-test. Both ergospirometry tests (i.e. with and without concurrent CLE) had identical settings and all tests were conducted by the same investigators within 7 days of each other.

Perceived exertion was quantified for all tests by use of the Borg rating of perceived exertion scale (RPE).

### Statistical analysis

Unpaired data were compared using unpaired t-test (normally distributed data) or Mann–Whitney U test (non-normally distributed data). Similarly, paired data were compared using paired t-tests or Wilcoxon’s signed rank test as appropriate. Test agreement was assessed using weighted kappa (*x*).[17,22] *P*-values lower than 0.05 were considered significant. Analysis was performed using SPSS (version 22.0; SPSS Inc., Chicago, IL, USA) and SAS 9.4 (SAS Institute Inc., Cary, NC, USA).

## Results

The 11 participants (10 women and one man) with EILO were aged mean (SD) 28 (23) years with a mean (SD) VO_2_max of 2608 (303) at the time of enrolment and a mean (SD) BMI of 24.4 (3.8). Asthma was present in 36% of the participants (Table 1).

**Table 1.** Participant characteristics.

Part.	Sex	Age in years	Height in centimetres	Weight in kilograms	FEV_1_% of pred.	FVC % of pred.	Co-morbidity
1	Female	21	164	59.50	120.6	119.5	No
2	Female	18	176	71.00	100	99.6	Asthma
3	Female	43	170	82.00	124.6	139.6	No
4	Female	29	165	62.00	105.9	137.8	Asthma
5	Female	33	177	75.20	105.1	125.6	No
6	Female	19	167	80.10	124.4	124.4	Asthma
7	Female	21	165	60.50	112.1	117.2	No
8	Female	21	167	61.60	125.8	120.6	No
9	Female	43	178	103.80	142.7	164.8	No
10	Female	25	170	59.20	86.1	85.7	Asthma
11	Male	37	173	66.10	99.5	123.3	No
Total	F: 91%/M: 9%	28(9)^a^	170 (5)^a^	71 (14)^a^	113 (16)^a^	123 (21) ^a^	Asthma: 36%

Part: participant.

F: female, M: male.

FEV_1_: forced expiratory volume in first second percentage of predicted.

FVC: forced expiratory volume percentage of predicted.

^a^ Numbers are mean (standard deviation)

The mean difference in maximal oxygen uptake measured by ergospirometry with and without simultaneous CLE testing was −22 ml O_2_･min^−1^ (95% CI −125 to 81 ml O_2_･min^−1^; *P *= 0.647) (Table 2). All tests performed both with and without CLE met the objective quality criteria for successful ergospirometry. The mean respiratory exchange ratio (RER) at peak exercise was 1.17 (95% CI 1.09 to 1.26) when measured with CLE and 1.24 (95% CI 1.15 to 1.32) when measured with ergospirometry alone (*P = *0.1) (Table 3); accordingly, there were no differences in maximum heart rates (*P = *0.6), maximum achieved watt (*P = *0.8), BORG RPE (*P *= 0.1) or test duration (*P *= 0.8).

**Table 2.** Ergospirometry and CLE results.

CLE, treadmill			Ergospirometry, bike				Ergospirometry with CLE, bike				
	Test duration	EILO	VO2max	%Pred VO2	Test duration	RPE	VO2max	%Pred VO2	Test duration	RPE	EILO
Part.	(sec)	G/SG	(ml·kg^–1^ ·min^–1^)		(sec)		(ml·kg^–1^·min^–1^)		(sec)		G/SG
1	270	0/2	41.9	97.4	495	17	46	107	515	19	0/2
2	255	0/2	35.1	79.6	520	19	38.5	87.4	510	19	0/2
3	270	0/2	30.4	87.2	432	18	33.4	95.8	415	18	0/2
4	210	1/2	40.2	100.4	465	19	44.1	110.3	420	19	0/2
5	260	0/2	33.1	85.9	480	18	36.4	94.4	490	19	0/2
6	186	2/3	31.1	71.1	515	20	34.2	78.1	510	20	1/3
7	245	0/2	41.2	95.8	475	20	45.2	105.2	490	20	0/2
8	270	2/2	40.4	94.1	505	19	44.4	103.3	510	20	1/2
9	240	0/2	24	68.9	540	19	26.4	75.7	575	19	1/3
10	375	2/3	42.1	101.4	500	19	46.2	111.4	460	19	2/2
11	360	2/0	37.7	101.7	575	20	41.4	111.7	560	20	0/0
Tot*^a^*	267.4		36.1	88.9	500.2	18	39.7	98.2	495.9	19.3	
	(56.2)		(5.9)	(11.9)	(38.4)	(0.9)	(6.5)	(13)	(50)	(0.6)	

CLE: Continuous laryngoscopy during exercise.

Part: participant.

Sec: seconds

EILO: exercise-induced laryngeal obstruction.

G/SG: glottic/supraglottic EILO.

VO2 max: maximum oxygen uptake.

RPE scale: Borg rating of perceived exertion scale.

*^a^* Tot: total. Numbers are mean (standard deviation).

**Table 3.** Ergospirometry measurements.

	Without CLE	With CLE	*P ^a^*
Maximum heart rate	191 (12)	191 (13)	0.6
Respiratory exchange ratio (RER)	1.24 (0.13)	1.17 (0.13)	0.1
Maximum watt achieved during test	194 (35.4)	203 (18.7)	0.8
Test duration in minutes	8.3 (0.6)	8.6 (1.5)	0.8
Maximal oxygen uptake in millilitres of O_2_ per minute	2608 (303)	2586 (308)	0.65

All values in this table are noted as mean (SD) unless otherwise stated.

*a*: paired t-tests

All CLE video recordings were of sufficient quality to determine EILO grade scores for both glottic and supraglottic obstruction. When comparing EILO scores obtained during the CLE testing on the treadmill with CLE testing on the ergometer bike, supraglottic scores (severity) were identical in nine of the 11 participants (82%) and there was substantial agreement between the two tests (*x* = 0.71), whereas glottic scores were identical in six of the 11 (55%), showing moderate agreement (*x* = 0.38) (Table 2).

### Patient acceptance of tests

In this study, no adverse events were seen despite two participants reporting mild discomfort and being anxious when initially fitting the mask during the trial ergospirometry test. The two participants successfully completed both the subsequent ergospirometry tests (with/without CLE).

## Discussion

To our knowledge, this study is the first to validate maximal oxygen uptake measurement (i.e. VO_2_max) obtained from ergospirometry with concurrent laryngoscopy. Although earlier reports exist on laryngoscopy on bike,[3,23] our study differs from these as our focus is on VO_2_max.

Our findings indicate that combining ergospirometry with CLE provides valid measurements of maximal oxygen uptake. Further, the current method provides supplementary measurements (Table 3) that not only confirm each acquired VO_2_max value (thus accounting for measurement errors),[19,24,25] but also hold potential predictive value [26] that should be further examined.

In contrast to Tervonen and colleagues,[27] we found that supraglottic EILO scores from the treadmill and ergometer bike were similar in severity, as they provoked identical disease severity in 82% of the observed cases, with a kappa value of *x* = 0.71. However, glottic scores were identical in only 55% of the observed cases, with a kappa value of *x* = 0.38. Our interrater agreement was moderate to substantial and similar to that of Røksund and colleagues [17] when examining the reliability of the current gold standard, i.e. continuous laryngoscopy during exercise on a treadmill. An explanation for these differences could be the lower frequency of vocal cord dysfunction (VCD) compared with EILO and the accordingly greater risk of false positive differences.

One case of VCD only (i.e. glottic EILO with no visible supraglottic EILO, Table 2) differed markedly between the test types: it was evaluated as a glottic score of 2 on the treadmill and zero on the bike. The CLE test on the treadmill detected glottic, supraglottic or a combined type of EILO in all cases, whereas on the bike, it detected EILO in only *n = *10 of these (91%). This, combined with the clinical assessment of all participants, suggests that the VO_2_max test with concurrent laryngoscopy has a greater specificity than the CLE test on the treadmill when detecting EILO in moderately active or inactive adults (Table 2).

EILO is often considered a condition restricted to elite athletes; however, this study shows that inactive and moderately active adults can also develop or have EILO, leading to clinically significant respiratory symptoms at a lower level of activity than seen in elite athletes. Accordingly, EILO is not a condition confined to elite athletes.

Our study supports the relevance of CLE *and* VO_2_max testing to better differentiate between respiratory-, cardiovascular- and/or inactivity-related limitations. The results of these tests provide objective measurements that can also aid clinicians in guiding patients when recommending lifestyle interventions.

As exhaustion is a key criterion of a successful VO_2_max test,[21] the use of an exercise bike instead of a treadmill has been questioned and assessed over the years [18,28] when testing athletes because their threshold of exhaustion differs between disciplines.[5] As this probably holds for all adults, we suggest that CLE should be performed in a sport-specific manner (when diagnosing a possible EILO). For example, a runner should perform the test on a treadmill, and a cyclist should perform the test on a bike. This is demonstrated in Panchasara and colleagues’ work [29] investigating rowing-induced laryngeal obstruction in elite rowers and by Walsted et al. [30] demonstrating the feasibility of CLE testing during swimming.[30]

As indicated in previous work,[29] our data support the theory that even though there is no difference in the usability of CLE tests on a bike and on a treadmill, when detecting EILO, these tests may differ according to the individual patient’s abilities.[23,31]

We also found that the laryngeal visualisations during exercise on the bike were superior to those on the treadmill: participants were stationary during exercise, giving a steadier video recording. Further, this set-up allowed the examiners to adjust the position of the laryngoscope during the test, which is not feasible during treadmill exercise laryngoscopy without interrupting the test. Overall, we found that CLE on the bike was reproducible, manageable and simply better for those participants who were better at cycling than running. As such, our findings support the earlier finding of Panchasara [29] suggesting the sense of having a lab set-up that allows patients to perform CLEs while doing the *specific activity* that causes their symptoms.

The present test combines two different examinations, and it was shown that the total testing time remains similar for CLEs conducted on a treadmill (approximately 8-min warm-up plus 5–6 min testing) and those on a bike (5-min warm-up plus 10 min testing); see Table 2.

## Strengths and limitations

Major strengths of this study were its randomised crossover design and the high quality of the tests with objective multiple criteria strictly dictating each test. Further, all our participants had a sedentary lifestyle; accordingly, our study deviates from earlier tests because those tests were performed in athletes. Our study was conducted prospectively and all patients referred to the outpatient asthma clinic of Bispebjerg for a CLE were evaluated for study inclusion. The foremost limiting factor of the study is the small sample size, not least concerning VCD, and the results concerning the glottis results might be underpowered. The small *n* is a common obstacle in EILO-related studies (and laryngology in general) and is thus generally accepted. Further work including a larger sample size is necessary to confirm and evaluate our findings, preferably in a setting allowing each participant to be evaluated during his/her preferred sport.

In summary, this randomised crossover comparison of ergospirometry measurements obtained with and without concurrent laryngoscopy in untrained individuals found ergospirometry with laryngoscopy to be feasible and well tolerated whilst yielding measurements for maximal oxygen uptake comparable to those obtained by standard ergospirometry.

The observed measurements of laryngeal closure were comparable to those obtained by the standard treadmill CLE test, verifying ergospirometry with concurrent laryngoscopy as a valid alternative to both treadmill CLE and ergospirometry, at least for supraglottic EILO.
